# Clinical data from the real world: efficacy of Crizotinib in Chinese patients with advanced ALK-rearranged non-small cell lung cancer and brain metastases

**DOI:** 10.18632/oncotarget.13179

**Published:** 2016-11-07

**Authors:** Puyuan Xing, Shouzheng Wang, Xuezhi Hao, Tongtong Zhang, Junling Li

**Affiliations:** ^1^ Department of Medical Oncology, Cancer Hospital, Chinese Academy of Medical Sciences (CAMS) and Peking Union Medical College (PUMC), Beijing, China

**Keywords:** NSCLC, crizotinib, brain metastasis, chinese, real world

## Abstract

Brain metastasis in non small cell lung cancer (NSCLC) patients is often considered as a terminal stage of advanced disease. Crizotinib is a small-molecule tyrosine kinase inhibitor (TKI) for ALK-rearranged NSCLC patients. Herein, we conducted a retrospective study to explore how Crizotinib affects the control of brain metastases and the overall prognosis in advanced ALK-rearranged NSCLC patients with brain metastases in Chinese population. A total of 34 patients were enrolled, of whom 20 (58.8%) patients had baseline brain metastases before Crizotinib treatment. Among patients with brain metastases before Crizotinib, overall survival (OS) after brain metastases was significantly longer than that of patients with brain metastases after Crizotinib (median OS, not reached vs. 10.3 months, respectively, *p* = 0.001). There was also a significant difference in systemic progression-free survival (PFS) between patients developing brain metastases before and after Crizotinib treatment (21.2 months vs. 13.9 months, *p* = 0.003). In conclusion, ALK-rearranged NSCLC patients with brain metastases before Crizotinib may benefit more from Crizotinib than those developing brain metastases during Crizotinib treatment.

## INTRODUCTION

Lung cancer has been the leading cause of cancer-related deaths both worldwide [1] and in China [2], among which 85% are non small cell lung cancer (NSCLC), mainly including adenocarcinoma and squamous cell carcinoma [3]. A large number of NSCLC patients have been at advanced stage when diagnosed and the main treatment option for these patients is platinum-based double-agent chemotherapy. However, traditional cytotoxic therapies have reached an efficacy plateau, with most of the patients who respond to them eventually progress [4]. Fortunately, breakthroughs in the molecular pathogenesis of NSCLC have facilitated the development of treatment targeting specific signaling pathways, and shed new light on NSCLC therapies.

Rearrangement of anaplastic lymphoma kinase (ALK) gene is also a potential target in NSCLC, the most common of which is EML4-ALK translocation [5]. The subsequent EML4-ALK fusion protein will be constitutively activated and transformed, activating the Ras and phosphoinositide 3-kinase (PI3K) signaling cascades [6], which might result in more aggressive tumor characteristics and possibly aggravated prognoses [7]. Crizotinib is a small-molecule tyrosine kinase inhibitor (TKI) of MET, ALK [8–10] and ROS1 [11, 12] kinases, which was approved for ALK-positive NSCLC by the US Food and Drug Administration in 2011 [13]. Several clinical studies have reported that Crizotinib led to improved outcomes [14, 15] and was extremely well tolerated [16].

However, the efficacy of Crizotinib in controlling systemic cancer has not effectively translated to the control of intracranial disease, with up to 60% of the patients developing brain metastases during the Crizotinib treatment [17], which may result from the poor central nervous system (CNS) penetration [18]. Hitherto, several case reports [18–30] and subset analyses of clinical trials [31] have evaluated the benefit of Crizotinib in controlling brain metastases, with various outcomes. These case reports cover patients of different characteristics, which make the outcomes unavailable to be compared. Herein, we conducted a retrospective analysis in advanced ALK-rearranged NSCLC patients, with the purpose of exploring how Crizotinib affects the control of brain metastases and the overall prognosis in the real world in an Asian population.

## RESULTS

### Baseline characteristics of patients

Baseline demographic and clinical characteristics of the enrolled patients are summarized in Table 1. A total of 34 patients were available for this retrospective study, of whom 30 (88.2%) patients presented with stage IV disease at diagnosis, 20 (58.8%) had baseline brain metastases at the initiation of Crizotinib treatment. Patients for this analysis were relatively young (median age, 51.5 years; range, 24–84 years), with 24 (70.6%) patients younger than 60 years old, and most of them (67.6%) were non-smokers. All patients were determined ALK translocation (19 by FISH, 13 by Ventana IHC test, and the others by RT-PCR). Meanwhile, 21 of all the patients were EGFR mutation negative, and the other patients' EGFR mutation status was unavailable. At the time of brain metastases development, patient ECOG PS was 0 (35.3% of patients), 1 (61.8% of patients), and 2 (2.9% of patients). After the brain metastases, 12 patients received local treatment (resection or radiotherapy) for brain metastases. A total of 27 patients (79.4%) developed extra-cranial metastases (ECM), including 12 bone metastases, 9 intrapulmonary metastases, 8 extra pulmonary lymph node metastases, 7 pleural metastases, 4 liver metastases and 2 mediastinal lymph node metastases.

**Table 1:** Patient demographic and clinical characteristics

	BM before the Treatment of Crizotinib		No BM before the Treatment of Crizotinib		All Enrolled Patients	
Characteristics	No.	%	No.	%	No.	%
Age, years						
Median	55		51		51.5	
Range	32–76		24–84		24–84	
Age distribution, years						
< 60	12	63.2	12	80.0	24	70.6
≥ 60	7	36.8	3	20.0	10	29.4
Sex						
Male	8	42.1	10	66.7	18	52.9
Female	11	57.9	5	33.3	16	47.1
Smoking history						
Yes	5	26.3	6	40.0	11	32.4
No	14	73.7	9	60.0	23	67.6
ECOG PS						
0	2	10.5	10	66.7	12	35.3
1	17	89.5	4	26.7	21	61.8
2	0	0	1	6.7	1	2.9
Extracranial metastases present						
Yes	7	36.8	15	100	22	64.7
No	12	63.2	0	0	12	35.3
Operation history						
Radical operation	6	31.6	5	33.3	11	32.4
Palliative operation	1	5.3	1	6.7	2	5.9
No	12	63.2	9	60.0	21	61.8

Abbreviations: BM, Brain Metastases; ECOG, Eastern Cooperative Oncology Group; PS, Performance Status.

### Treatment, follow-up and disease control with Crizotinib

Patients receiving Crizotinib at the first, second or ≥ third line of treatment were 9 (26.5%), 16 (47.1%) and 9 (26.5%), respectively. Twenty-five patients were treated with chemotherapy at the first line, among which 15 (60.0%) with Pemetrexed-based, 2 (8.0%) with Gemcitabine-based and 8 (32.0%) with Paclitaxel-based regimens. Median follow-up was 16.1 months.

**Figure 1:**
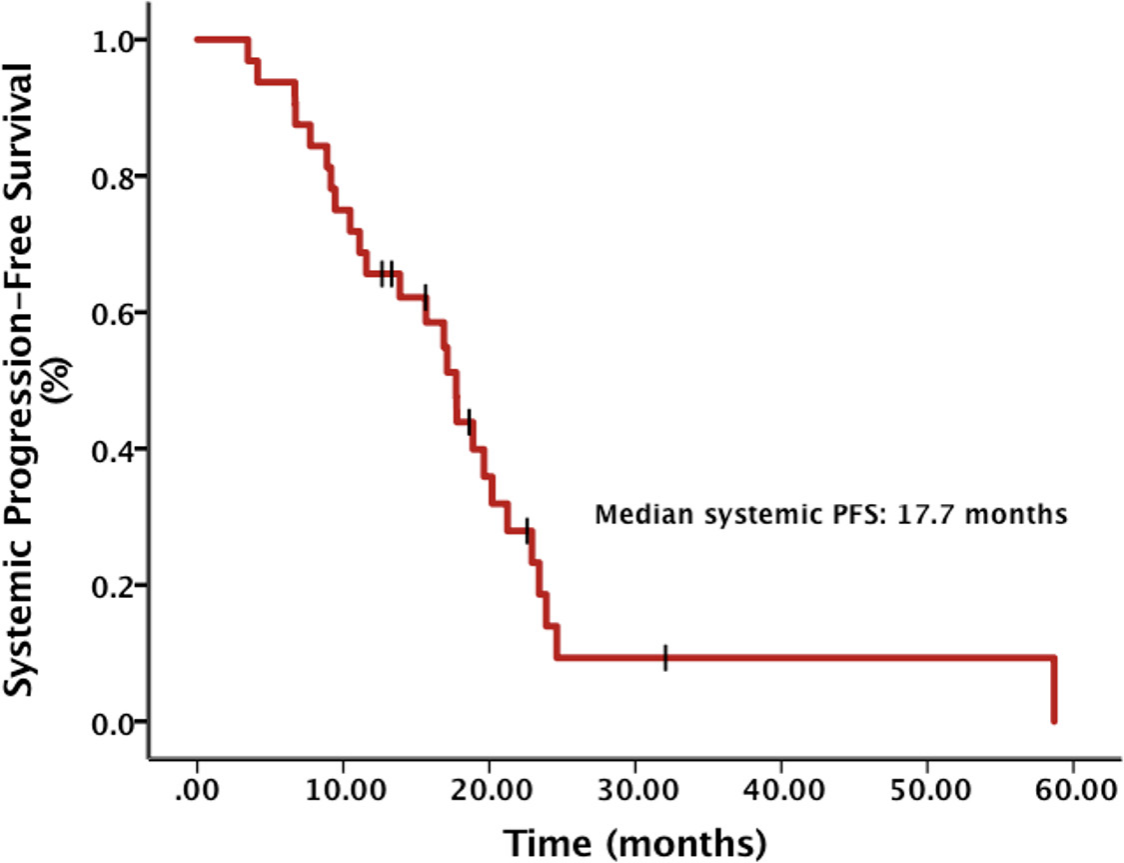
Systemic progression-free survival (PFS) from diagnosis of advanced NSCLC

**Figure 2:**
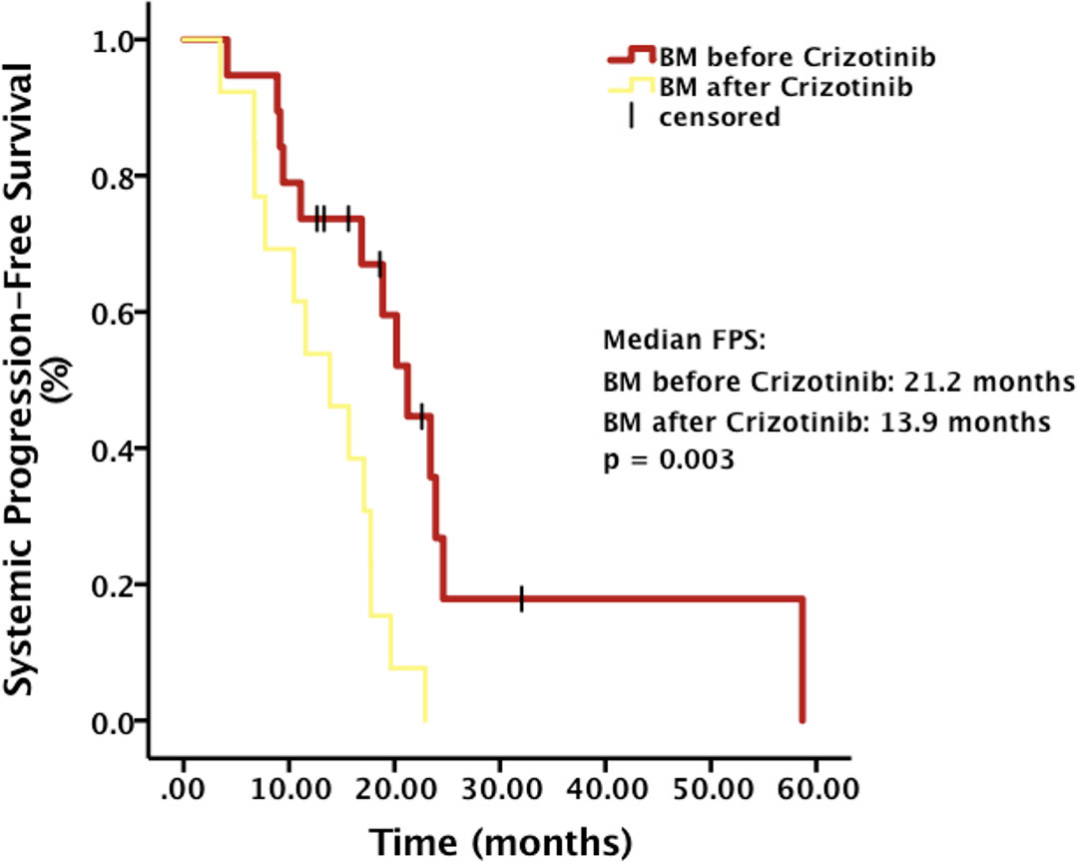
Systemic progression-free survival (PFS) from diagnosis of advanced NSCLC stratified by baseline brain metastases (BM) status

The objective response rate (ORR) of all patients was 29.4% and the brain lesion ORR of patients with brain metastases before Crizotinib was 15.0%. The median systemic progression-free survival (PFS) calculated from the diagnosis of advanced NSCLC was 17.7 months (95% CI, 15.2 – 20.3) (Figure 1). There was a significant difference in systemic PFS between patients developing brain metastases before and after Crizotinib treatment (21.2 months vs. 13.9 months, *p* = 0.003) (Figure 2). The median intracranial PFS (IC-PFS) among patients with brain metastases before Crizotinib was 20.7 months (95% CI, 17.8 – 23.6). The median time to brain metastases from diagnosis of advanced NSCLC among patients with brain metastases after Crizotinib was 13.4 months (95% CI, 5.9 – 20.9).

**Figure 3:**
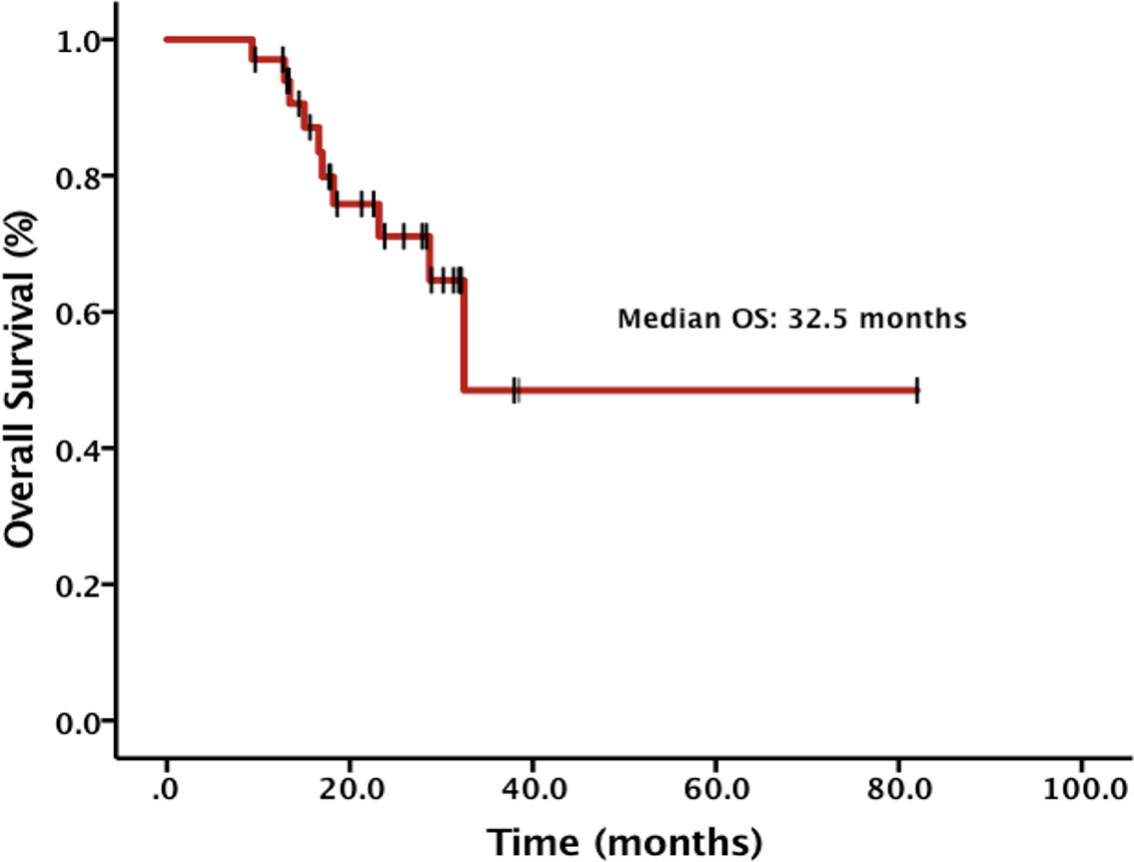
Overall survival (OS) from diagnosis of advanced NSCLC

The median OS from diagnosis of advanced NSCLC was 32.5 months (Figure 3), with 10 patients died (29.4%). 1- and 2-year overall survival rates were 97.1% and 71.1%, respectively. There's no significant difference between the median OS of patients with or without extra-cranial metastases (32.5 months vs. not reached, *p* = 0.431). The median OS after brain metastases was not reached and the 1- and 2-year OS rates were 76.2% and 64.9%, respectively.

**Figure 4:**
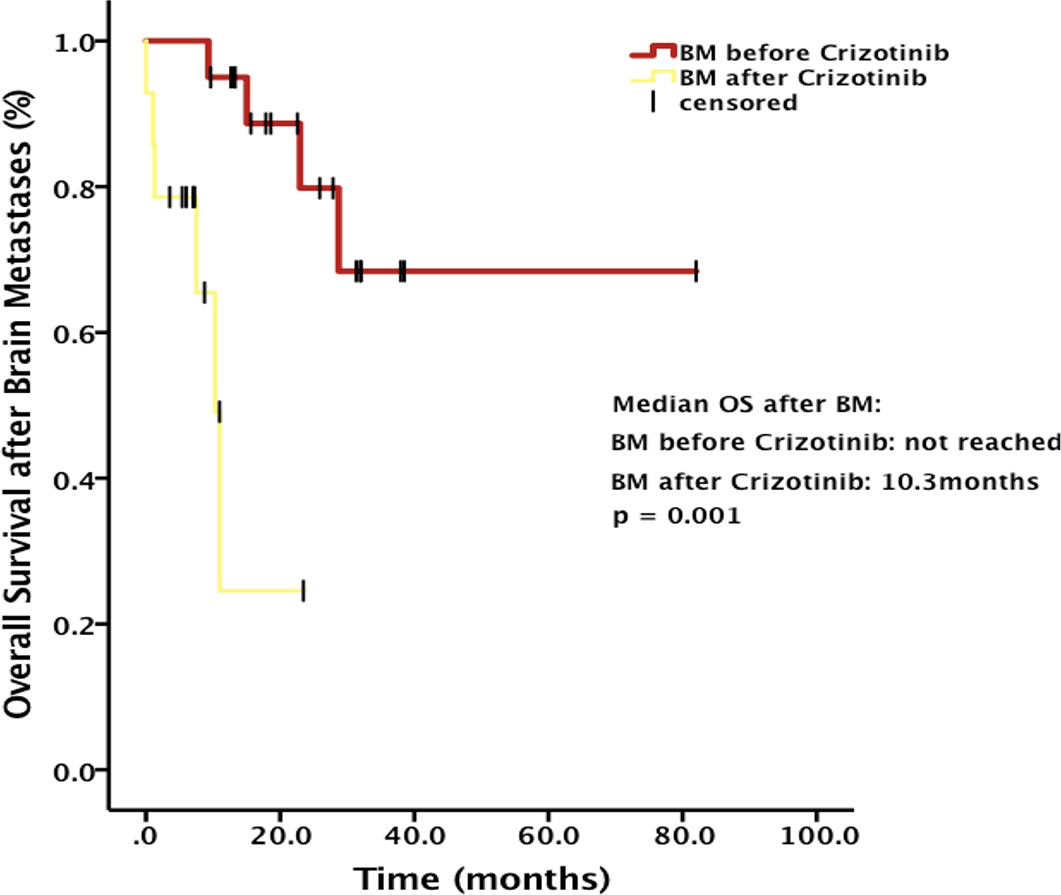
Overall survival (OS) after brain metastases (BM) stratified by baseline BM status

For patients treated with Crizotinib after brain metastases, OS after brain metastases was significantly longer, compared with patients developing brain metastases during Crizotinib treatment (median OS, not reached vs. 10.3 months, respectively, *p* = 0.001) (Figure 4). Either in patients receiving local treatment or not, there was a significant difference in OS after brain metastases between patients developing brain metastases before and after Crizotinib treatment (*p* = 0.042 and 0.03, respectively).

Among all the patients treated with chemotherapy at the first line, OS after brain metastases in patients with baseline brain metastases before Crizotinib was significantly superior to that in patients developing brain metastases after Crizotinib (*p* = 0.023). Whereas, among patients receiving Crizotinib at the first line, OS after brain metastases in patients with baseline brain metastases before Crizotinib didn't demonstrate such superior, compared with patients without baseline brain metastases (*p* = 0.089).

**Figure 5:**
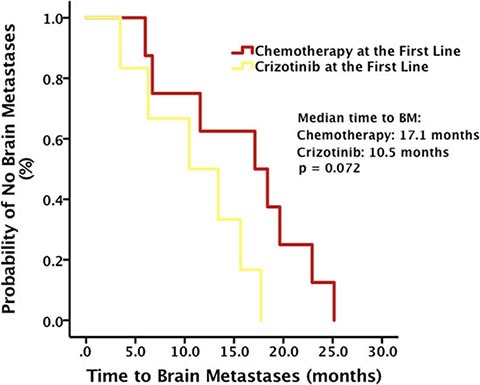
Time to brain metastases (BM) stratified by the first line treatment

Among patients who developed brain metastases during Crizotinib administration, though the result was not significant by the cut-off date, time to brain metastases, calculated from the diagnosis of advanced NSCLC, was longer in patients receiving chemotherapy at the first line, compared with that in patients receiving Crizotinib at the first line (median time to brain metastases, 17.1 months vs. 10.5 months, *p* = 0.072) (Figure 5).

### Adverse events and dose modification

Crizotinib related adverse events (AE) that were found in > 5% of patients are summarized in Table 2. The most common AEs of all grades (in > 30% of all the enrolled patients) include cardiac disturbances (CK-MB elevation, 62.9%), hepatic disturbances (ALT increase, 42.9%) and endocrine disruptions (hypocalcemia, 34.3%). Dose reductions or temporary modifications were required in four patients due to the severe adverse reactions (one for prolonged QTc interval, one for hepatic disturbance, one for diarrhea and the other for hypocalcemia).

**Table 2:** Crizotinib related adverse events

	BM before the Treatment of Crizotinib		No BM before the Treatment of Crizotinib		All Enrolled Patients	
Adverse Events	All Grades	Grade ≥ 3	All Grades	Grade ≥ 3	All Grades	Grade ≥ 3
Cardiac disturbances						
CK-MB elevation	13 (68.4%)	0	9 (56.3%)	0	22 (62.9%)	0
Peripheral edema	6 (31.6%)	0	0	0	6 (17.1%)	0
QTc prolongation	1 (5.3%)	0	2 (12.5%)	0	3 (8.6%)	0
Hepatic disturbances						
ALT increase	10 (52.3%)	1 (5.3%)	5 (31.3%)	0	15 (42.9%)	1 (2.9%)
Endocrine disruptions						
Hypocalcemia	6 (31.6%)	0	6 (37.5%)	0	12(34.3%)	0
Bone marrow depression						
Neutropenia	5 (26.3%)	0	1 (6.3%)	1(6.3%)	6 (17.1%)	1 (2.9%)
Gastrointestinal disturbances						
Nausea/vomiting	4 (21.1%)	0	1 (6.3%)	0	5 (14.3%)	0
Diarrhea	3 (15.8%)	0	1 (6.3%)	0	4 (11.4%)	0
Constipation	2 (10.5%)	0	2 (12.5%)	0	4 (11.4%)	0
Ocular disturbances						
Diplopia	2 (10.5%)	0	1 (6.3%)	0	3 (8.6%)	0

Abbreviations: BM, Brain Metastases; CK-MB, Creatine Kinase-MB; ALT, Alanine Aminotransferase.

## DISCUSSION

Brain metastasis in NSCLC patients, which develops in 15% to 40% of ALK-rearranged patients after first diagnosis [32–34] and in approximately one third after the failure of at least one prior systemic therapy [31], is often considered as the terminal stage of advanced disease. Crizotinib is thought to be a small-molecule ALK inhibitor with poor penetration of blood-brain barrier. A subset analysis of clinical trials PROFILE 1005 and 1007 has reported [31] that in ALK-rearranged advanced NSCLC patients treated with Crizotinib, the intracranial disease control rate (DCR) and ORR at 12 weeks were 56% (62%) and 18% (33%), with a median intracranial time to progression (TTP) of 7 months (13.2 months) in patients with previously untreated (treated) brain metastases, respectively. Lei YY et al. [35] has reported a similar ORR of Crizotinib between patients with and without baseline brain metastases (68.4% vs. 69.5%, *p* = 0.904) and a significantly longer median PFS of patients without baseline brain metastases (10.0 months vs. 7.0 months, *p* = 0.021). However, a mature overall survival of these patients are still rudimentary.

To our knowledge, our retrospective study of Crizotinib in patients with advanced ALK-rearranged NSCLC and brain metastases represents the first and the largest data focusing on the overall prognosis of Chinese population in the real world.

In our study, the systemic ORR of all the patients was 29.4% and the brain lesion ORR of patients with brain metastases before Crizotinib was 15.0%, which seemed to be obviously inferior to the results of clinical trials PROFILE 1005 and 1007. However, 1- and 2-year OS rates (76.2% and 64.9%) in our study were similar to those observed by Johung KL et al. (72% and 66%) in a multi-institutional study which provided the largest data set of long-term outcomes for ALK-rearranged NSCLC patients with brain metastases in USA [36].

Our analyses suggested that among patients with baseline brain metastases before Crizotinib treatment, OS after brain metastases was significantly longer, compared with patients without brain metastases before Crizotinib treatment (median OS, not reached vs. 10.3 months, *p* = 0.001). There was also a significant difference in systemic PFS between patients developing brain metastases before and after Crizotinib treatment (21.2 months vs. 13.9 months, *p* = 0.003). In Johung KL et al. study, they contributed such difference to the control of CNS disease with radiotherapy. In our study, we also calculated the OS after brain metastases stratified by brain local treatment. Either in patients receiving local treatment or not, there was a significant difference in OS after brain metastases between patients developing brain metastases before and after Crizotinib treatment (*p* = 0.042 and 0.03, respectively). This result excluded the brain local treatment as a confounding factor of the OS in our study.

Furthermore, to investigate whether the first-line treatment had impact on the OS after brain metastases, we analyzed OS stratified by first-line treatment as well. Among all the patients treated with chemotherapy at the first line, OS after brain metastases in patients with brain metastases before Crizotinib was significantly superior to that in patients developing brain metastases after Crizotinib (*p* = 0.023). Such result indicated that these patients with brain metastases could still benefit from Crizotinib after first-line chemotherapy failure. Acquired resistance of Crizotinib may partly explain the result, and brain metastases during the Crizotinib treatment might be a strong signal of such resistance. Several studies have discussed about the mechanisms of such acquired resistance. The major mechanisms include ALK tyrosine kinase mutations, ALK copy number gain, activation of bypass signaling, and manipulation of downstream signaling pathways [37–41]. For patients with Crizotinib resistance, one promising therapeutic approach is second-generation ALK TKIs, such as Ceritinib, Alectinib and Brigatinib. Ceritinib, with more effective diffusion through blood-brain barrier, has demonstrated significant CNS activity in patients receiving prior Crizotinib, with a median intracranial duration of response time of 12.8 months [42].

Whereas, among patients receiving Crizotinib at the first line, OS after brain metastases in patients with baseline brain metastases before Crizotinib didn't demonstrate such superior, compared with patients without baseline brain metastases (*p* = 0.089). Moreover, even though the result was not significant by the cut-off date, time to brain metastases was longer in patients receiving chemotherapy at the first line, compared with patients receiving Crizotinib at the first line (*p* = 0.072). These results suggested that chemotherapy would be a better choice than Crizotinib for preventing intracranial progression. One probable reason for these findings might be associated with the poor penetration rate of Crizotinib into the cerebrospinal fluid (CSF). It was reported that the plasma concentration of Crizotinib was measured at 237 ng/mL, while the CSF concentration was 0.616 ng/mL, with a CSF-to-plasma ratio of 0.0026 [18].

There were several limitations for our current study. On the one hand, as a retrospective study involving patients from one cancer center, bias may have been introduced by the patient cohort. On the other hand, because of the relatively short follow-up time, we could see the trend that time to brain metastases was longer in patients receiving chemotherapy at the first line, compared with patients receiving Crizotinib, but the current result was not significant. Thus, a multicenter prospective study would be required to further confirm our results.

In conclusion, our data suggested that ALK-rearranged NSCLC patients with baseline brain metastases may benefit more from Crizotinib than those developing brain metastases during Crizotinib treatment.

## MATERIALS AND METHODS

### Patients

We retrospectively investigated advanced NSCLC patients with brain metastases who underwent Crizotinib treatment at the Cancer Hospital of the Chinese Academy of Medical Sciences (Beijing, China) between April 2013 and October 2015. Patients meeting the following criteria were included: having a pathological diagnosis of NSCLC, developing brain metastases either before or during the treatment of Crizotinib, having treated with Crizotinib at any line of treatment. ALK translocation was determined by Ventana IHC test, FISH or RT-PCR. Brain metastases were diagnosed by CT or MRI. In this study, Crizotinib was administered at a dose of 250 mg twice daily, with proper adjustments as needed. The follow-up was done by regular visits or telephone calls, and the information was collected into our database for analyses.

### Data extraction

Baseline characteristics were recorded, including age, sex, stage at diagnosis, smoking history, ECOG PS scores, presence of extra-cranial metastases and operation history. Treatment dates, follow-up, treatment at the first line, presence of brain metastases at the initiation of Crizotinib treatment, dates of developing brain metastases, and dates of systemic or brain disease progression were also recorded. Adverse events were reported based on the National Cancer Institute Common Terminology Criteria for Adverse Events (CTCAE, version 3.0).

### Statistical analyses

Statistical analyses were carried out by the SPSS 23.0 statistical software (SPSS, Inc., Chicago, IL, USA). Systemic OS and PFS after diagnosis of NSCLC were analyzed with Kaplan-Meier method. Kaplan-Meier analysis was also used to calculate OS after brain metastases, PFS and time to brain metastases stratified by patients or treatment characteristics. The median event time and corresponding 95% CI were also provided. The differences were assessed by the log-rank test. Statistical tests were two-sided, and *p* < 0.05 was considered significant.
